# Clinical Decision Support Systems to Predict Drug–Drug Interaction Using Multilabel Long Short-Term Memory with an Autoencoder

**DOI:** 10.3390/ijerph20032696

**Published:** 2023-02-02

**Authors:** Fadwa Alrowais, Saud S. Alotaibi, Anwer Mustafa Hilal, Radwa Marzouk, Heba Mohsen, Azza Elneil Osman, Amani A. Alneil, Mohamed I. Eldesouki

**Affiliations:** 1 Department of Computer Sciences, College of Computer and Information Sciences, Princess Nourah bint Abdulrahman University, P.O. Box 84428, Riyadh 11671, Saudi Arabia; 2 Department of Information Systems, College of Computing and Information System, Umm Al-Qura University, Makkah 24211, Saudi Arabia; 3 Department of Computer and Self Development, Preparatory Year Deanship, Prince Sattam bin Abdulaziz University, Al Kharj 16436, Saudi Arabia; 4 Department of Information Systems, College of Computer and Information Sciences, Princess Nourah bint Abdulrahman University, P.O. Box 84428, Riyadh 11671, Saudi Arabia; 5 Department of Computer Science, Faculty of Computers and Information Technology, Future University in Egypt, New Cairo 11835, Egypt; 6 Department of Information System, College of Computer Engineering and Sciences, Prince Sattam bin Abdulaziz University, Al Kharj 16436, Saudi Arabia

**Keywords:** healthcare, decision making, big data, drug–drug interaction, deep learning, predictive models

## Abstract

Big Data analytics is a technique for researching huge and varied datasets and it is designed to uncover hidden patterns, trends, and correlations, and therefore, it can be applied for making superior decisions in healthcare. Drug–drug interactions (DDIs) are a main concern in drug discovery. The main role of precise forecasting of DDIs is to increase safety potential, particularly, in drug research when multiple drugs are co-prescribed. Prevailing conventional method machine learning (ML) approaches mainly depend on handcraft features and lack generalization. Today, deep learning (DL) techniques that automatically study drug features from drug-related networks or molecular graphs have enhanced the capability of computing approaches for forecasting unknown DDIs. Therefore, in this study, we develop a sparrow search optimization with deep learning-based DDI prediction (SSODL-DDIP) technique for healthcare decision making in big data environments. The presented SSODL-DDIP technique identifies the relationship and properties of the drugs from various sources to make predictions. In addition, a multilabel long short-term memory with an autoencoder (MLSTM-AE) model is employed for the DDI prediction process. Moreover, a lexicon-based approach is involved in determining the severity of interactions among the DDIs. To improve the prediction outcomes of the MLSTM-AE model, the SSO algorithm is adopted in this work. To assure better performance of the SSODL-DDIP technique, a wide range of simulations are performed. The experimental results show the promising performance of the SSODL-DDIP technique over recent state-of-the-art algorithms.

## 1. Introduction

In the digital era, the velocity and volume of public, environmental, health, and population data from a wider variety of sources are rapidly developing. Big Data analytics technologies such as deep learning (DL), statistical analysis, data mining (DM), and machine learning (ML) are used to create state-of-the-art decision models [1]. Decision making based on concrete evidence is crucial and has a dramatic effect on program implementation and public health. This highlights the significant role of a decision model under uncertainty, involving health intervention, disease control, health services and systems, preventive medicine, quality of life, health disparities and inequalities, etc. A drug–drug interaction (DDI) can occur when more than one drug is co-prescribed [2]. Even though DDIs might have positive impacts, sometimes they have serious negative impacts and result in withdrawing a drug from the market. DDI prediction could assist in reducing the possibility of adverse reactions and improve the post-marketing surveillance and drug development processes [3]. Medical trials are time consuming and impracticable with respect to dealing with largescale datasets and the limitations of experimental conditions. Hence, researchers have presented a computation method to speed up the process of prediction [4]. The present computation DDI prediction method is divided into five classes of models: DL-based, network-based, similarity-based, literature extraction-based, and matrix factorization-based models.

ML techniques are an emerging area which are employed in large datasets for extracting hidden concepts and relationships amongst attributes [5]. An ML model can be used to forecast outcomes. Since it is extremely complex for humans to process and handle a large amount of data [6], hence, an ML model can play a major role to forecast healthcare outcomes with high quality and cost minimization [7]. ML algorithms are based primarily on rule-based, probability-based, tree-based, etc. methods. Large quantities of data gathered from a variety of sources are applied in the data preprocessing stage. During this stage, data dimension is minimized by eliminating redundant data. As the amount of data increases, a model is not capable of making a decision. Hence, various methods must be developed so that hidden knowledge or useful patterns are extracted from previous information [8]. Then, a model using a ML algorithm is tested under test data to discover the model’s performance, which can be augmented again by considering some rules or parameters. Generally, ML is utilized in the area of prediction, data classification, and pattern recognition [9]. Numerous applications such as disease prediction, face detection, fraud detection, traffic management, and email filtering, use the ML concept. The DL method is part of ML algorithms, which makes use of supervised and unsupervised models for feature classification [10]. The various elements of DL approaches are utilized in the field of recommender systems, disease prediction, and image segmentation such as restricted Boltzmann machines (RBM), convolution neural networks (CNN), and autoencoders (AEs).

In this study, we develop a sparrow search optimization with deep learning-based DDI prediction (SSODL-DDIP) technique for healthcare decision making in big data environments. The presented SSODL-DDIP technique applies a multilabel long short-term memory with an autoencoder (MLSTM-AE) model for the DDI prediction process. Moreover, a lexicon-based approach is involved in determining the severity of interactions among the DDIs. To improve the prediction outcomes of the MLSTM-AE model, the SSO algorithm is adopted in this work. For ensuring better performance of the SSODL-DDIP technique, a wide range of simulations are performed.

## 2. Related Works

In [10], the authors proposed a positive unlabeled (PU) learning model which utilized a one-class support vector machine (SVM) model as the learning algorithm. The algorithm could learn the positive distribution from the unified feature vector space of drugs and targets, and regarded unknown pairs as unlabeled rather them labeling them as negative pairs. Wang et al. [11] introduced a novel technique, multi-view graph contrastive representative learning for DDI forecasting, MIRACLE for brevity, for capturing intra-view interactions and inter-view molecular structure among molecules concurrently. MIRACLE treated a DDI network as a multi-view graph in which all nodes in the interaction graph were a drug molecule graph sample. The author employed a bond-aware attentive message propagating algorithm for capturing drug molecular structured data and a graph convolution network (GCN) for encoding DDI relations in the MIRACLE learning phase. Along with that, the author modeled an innovative unsupervised contrastive learning element to integrate and balance multi-view data. In [12], the author devised a deep neural networks (DNNs) method that precisely identified the protein–ligand interactions with particular drugs. The DNN could sense the response of protein–ligand interactions for the particular drugs and could find which drug could effectively combat the virus.

Lin et al. [13] modeled an end-to-end structure, named a knowledge graph neural network (KGNN), for resolving DDI estimation. This structure could capture a drug and its neighborhood by deriving their linked relations in a knowledge graph (KG). For extracting semantic relations and high-order structures of the KG, the author studied the neighborhoods for all entities in KG as its local receptive, and then compiled neighborhood data from representations of the current entities. Pang et al. [14] presented a new attention-system-related multidimensional feature encoder for DDI estimation, called attention-related multidimensional feature encoders (AMDEs). To be specific, in an AMDE, the author encoded drug features from multidimensional features, which included data from an atomic graph of the drug and a simplified molecular-input line-entry system sequence. Salman et al. [15] modeled a DNN-oriented technique (SEV-DDI: Severity-DDI) that included certain integrated units or layers for attaining higher accuracy and precision. The author moved a step further and used the techniques for examining the seriousness of the interaction, after outpacing other methods in the DDI classifier task successfully. The capability to determine DDI severity helps in clinical decision aid mechanisms for making very precise and informed decisions, assuring the patient’s safety.

Liu et al. [16] presented a deep attention neural network-related DDI predictive structure (DANN-DDI), for forecasting unnoticed DDIs. Firstly, by utilizing the graph embedding technique, the author framed multiple drug feature networks and learned drug representation from such networks; after that, the author concatenated learned drug embeddings and implemented an attention neural network for learning representation of drug-drug pairs; finally, the author devised a DNN to precisely estimate DDIs. Zhang et al. [17] introduced a sparse feature learning ensembled approach with linear neighborhood regularization (SFLLN), for forecasting DDIs. Initially, the authors compiled four drug features, i.e., pathways, chemical structures, enzymes, and targets, by mapping drugs in distinct feature spaces into general interaction spaces by sparse feature learning. Then, the authors presented the linear neighborhood regularizations for describing the DDIs in the communication space by utilizing known DDIs. 

## 3. The Proposed Model

In this study, we introduce a novel SSODL-DDIP technique for DDI predictions in big data environments. The presented SSODL-DDIP technique accurately determines the relationship and drug properties from various sources to make predictions. It encompasses data preprocessing, MLSTM-AE-based DDI prediction, SSO hyperparameter tuning, and severity extraction.

### 3.1. Data Preprocessing

Standard text cleaning and preprocessing operations were carried out on sentences involving but not constrained to lemmatization. Every drug discussed in a sentence was considered and labeled to interact with others [18]. The number of drug pairs (DP) in a sentence is evaluated as follows:

(1) $$Drug\;Pairs\;\left(DP\right)=\;\max\;\left(0,\overset{n}{\underset{i=1}{\sum }}(i-1\right))$$

where n indicates the number of drugs in a sentence.

In addition, drug blinding was used, whereby all the drug names were allocated to the label, for a sentence, “Aspirin might reduce the effect of probenecid”, labeled sentence was “$Drug^{A}$ might reduce the effect of $Drug^{B}$”. The drug blinding method assists a technique to identify this label as ”subject” and ”object” that ultimately assist an approach during classification. Then, the processed sentence is given to the approach for classification and detection of DDI.

During word embedding, every word was converted into a real value vector. This word mapping into the matrix can be performed using Word2Vec and embedding data using the abstract of PubMed comprising the drugs.


(2) $$s_{i}^{\to }=W_{EMB}\cdot v_{i}^{s}$$

Every sentence is preprocessed and constitutes “$s_{i}$” and “$d_{j}$”, where $d_{j}$ represents drug labels and $s_{i}$ is another word in the sentence. Every word “$s_{i}$” is transformed to the word vectors using the word embedding matrices. Word embedding (WEMB) is an embedding matrix and WEMB $\in {\mathbb{R}}^{d_{s}\times \left|V\right|}$ whereas $V^{\prime }$ denotes the vocabulary in the training dataset, $d_{s}$ signifies the count of dimensions, and $v_{i}^{s}$ denotes the index of word embedding.

### 3.2. DDI Prediction Process

**Figure 1:**
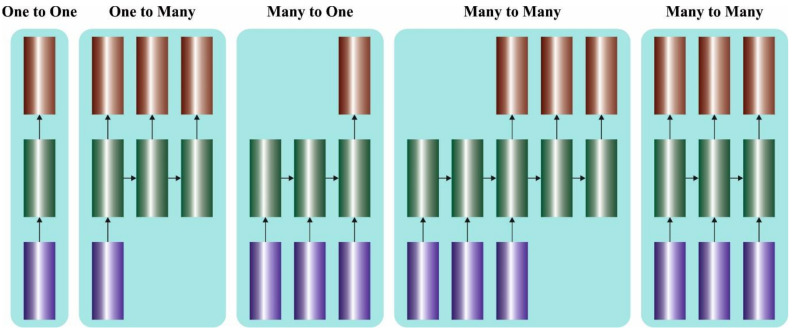
Structure of MLSTM.

To predict the DDI accurately, the MLSTM-AE model is applied in this study. The MLSTM-AE model learns to recreate a time flipped version of input [19]. Every input electricity signal is denoted as $\chi_{i}=\left\{\chi_{i}^{1},\chi_{i}^{2},\ldots ,\chi_{i}^{T}\right\}$, and is of length T. The hidden state vectors of long short-term memory (LSTM) encoding at $P^{h}$ instant are represented as $h_{i}^{t}$. The encoder captures relevant data to recreate the input signals. Once it encodes the final point in the input, the $h_{i}^{T}$ hidden state of the encoder is the vector depiction for the input $\chi_{i}$. The decoding has a similar network architecture as the encoding; however, it learns to recreate a flipped version of e input, viz., $\left\{\chi_{i}^{T},\;\chi_{i}^{T-1},\;\chi_{i}^{1}\right\}$. The last hidden state $h_{i}^{T}$ of the encoder can be utilized as the first hidden state of decoding input. The targeted output acts as a flipped version of input, viz., $\left\{\chi_{i}^{T},\chi_{i}^{T-1},\ldots ,\chi_{i}^{1}\right\}$ and the actual recreated one $\mathrm{is}\;\left\{\hat{\chi },\hat{\chi },\ldots ,\hat{\chi }\right\}$. The presented method has been demonstrated. Now, the encoder and the decoders are LSTM for modeling dynamic signals. The depiction from the deep layer of the encoder is interconnected with the output label through fully connected networks (FCNs). The reconstruction utilized for training the MLSTM-AE model is formulated by:

(3) $$L_{rec}=\frac{1}{N}\overset{N}{\underset{i=1}{\sum }}\overset{T}{\underset{;=1}{\sum }}{\left(x_{i}^{t}-{\hat{x}}_{i}^{t}\right)}^{2}$$

where N denotes the overall sample count. Because, the final objective of the study is to learn to categorize, the embedding from the $h_{i}^{T}$ hidden layer is passed via a fully connected (FC) layer, the output of which is the class label. The class label is one-hot encoded. The size of the label vector is equivalent to the number of appliances; once an appliance is ON, the corresponding location of the label vector is 1 or else 0. This can be denoted by $y_{i}=\left\{y_{i}^{1},y_{i}^{2},\;\ldots ,y_{i}^{c}\right\},$ considering C appliances. Figure 1 represents the structure of MLSTM.

When the $C^{th}$ appliance is ON, the corresponding $y_{i}^{c}$ is 1; otherwise it is 0. The ground-truth probability vector of $i^{th}$ samples are described as ${\hat{p}}_{i}=y_{i}/\|y_{i}{\|}_{1}$. The predicted probability vector can be represented as $p_{i}$.


(4) $$L_{cls}=\frac{1}{N}\overset{N}{\underset{i=1}{\sum }}\overset{c}{\underset{c=1}{\sum }}{\left(p_{i}^{c}-{\hat{p}}_{i}^{c}\right)}^{2}$$

This algorithm has been trained collectively with the reconstruction loss and multilabel classification loss, hence, the overall loss function is formulated by Equation (5):


(5) $$L=L_{cls}+\gamma L_{rec}$$

### 3.3. Hyperparameter Tuning Process

For the hyperparameter tuning process, the SSODL-DDIP technique uses the SSO algorithm. The SSO is a recent metaheuristic approach which stimulates the anti-predatory and predation actions of the sparrow population [20], particularly, in foraging, individual sparrows act in two roles: joiner and discoverer. The discoverer is responsible for searching the food and guiding others, and the joiner forages by following the discoverers. A specific percentage of sparrows has been carefully chosen as the guarder that transmits alarm signals and carries out anti-predation behavior while they realize the danger. The discoverer position can be redeveloped as follows:


(6) $$X_{i,j}^{t+1}=\{\begin{array}{ll} X_{i,j}^{t}\cdot \;\exp\;\left(-\frac{i}{\alpha \cdot T}\right) & R_{2}<ST\\ X_{i,j}^{t}+O\cdot G & R_{2}\geq ST \end{array}$$

In Equation (6), t is the existing value of update. T presents the maximal value of update. $X_{ij}^{t}$ defines the present position of the i-th agent. $X_{ij}^{t+1}$ denotes the upgraded position of the i-th sparrow in the j-th dimension α∈(0,1] refers to a random number. ST∈(0.5,1] signifies a safety value. $R_{2}\in (0,1$] defines a warning value. G denotes a 1×d matrix where each value is 1. O represents a random variable.

The joiner position is regenerated as follows:


(7) $$X_{i,j}^{t+1}=\{\begin{array}{ll} O\cdot \;\exp\;\left(\frac{x_{w}-X_{i,j}^{t}}{i^{2}}\right) & i>n/2\\ X_{b}+\left|X_{i,j}^{t}-X_{b}\right|\cdot B\cdot G & otherwise \end{array}$$

**Figure 2:**
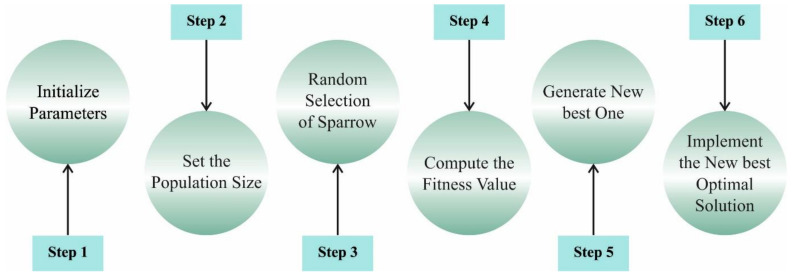
Steps involved in the SSO algorithm.

In Equation (7), $X_{b}$ signifies the existing optimum position of the discoverer. $X_{w}$ describes the worst position of the sparrow, B denotes the 1×d matrix where every value is equivalent to 1 or −1, and $A^{+}=A^{T}{\left(AA^{T}\right)}^{-1}$. Figure 2 demonstrates the steps involved in the SSO algorithm.

The position regeneration for the guarder can be defined as follows:


(8) $$X_{i,j}^{t+1}=\{\begin{array}{ll} X_{best}^{t}+\beta \cdot \left|X_{i,j}^{t}-X_{best}^{t}\right| & ft_{j}>ft_{g}\\ X_{i,j}^{t}+K\cdot \left(\frac{X_{i,i}^{t}-X_{worst}^{t}}{(f^{t_{i}}-f^{t_{w})+\varepsilon }}\right) & ft_{i}=ft_{g} \end{array}$$

In Equation (8), $X_{best}$ stand for the best global location. β and K∈[−1,1] represent two random integers; $ft_{i}$ defines the fitness value. $ft_{w}$ and $ft_{g}$ are the present worst and best fitness values in the population, correspondingly; ε indicates a minimal number that is closer to zero as explained in Algorithm 1.
**Algorithm 1:** Pseudocode of SSO algorithmDefine $Iter_{\max}$, $NP,\;n,\;P_{dp},\;sf,\;G_{c},\;FS_{U}$ and $FS_{L}$ Arbitrarily initializing the flying squirrels places
$$FS_{i,j}=FS_{L}+rand()*\left(FS_{U}-FS_{L}\right),\;i=1,2,\;\ldots ,\;NP,\;j=1,2,\;\ldots ,\;n$$
Compute fitness value
$$f_{i}=f_{i}\left(FS_{i,1},FS_{i,2},\ldots ,\;FS_{i,n}\right),\;i=1,2,\;\ldots ,\;NP$$
while $Iter<Iter_{\;\max\;}$
[sorted‐f, sorte‐index] =sort(f)
$$FS_{ht}=FS\left(sorte\_index\left(1\right)\right)$$
$$FS_{at}\left(1:3\right)=FS\left(sorte_{-}index\left(2:4\right)\right)$$
$$FS_{nt}\left(1:NP-4\right)=FS\left(sorte\_index\left(5:NP\right)\right)$$
Create novel places
for t=1:n1(n1= entire count of squirrels on acorn trees)
    if $R_{1}\geq P_{dp}$
$$FS_{at}^{new}=FS_{at}^{old}+d_{g}G_{c}\left(FS_{ht}^{old}-FS_{at}^{old}\right)$$
    else
$$FS_{at}^{new}=random\;location$$
    end
end
for t=1:n2(n2= entire count of squirrels on normal trees moving to acorn trees)
    if $R_{2}\geq P_{dp}$
$$FS_{nt}^{new}=FS_{nt}^{old}+d_{g}G_{c}\left(FS_{at}^{old}-FS_{nt}^{old}\right)$$
    else
$$FS_{nt}^{new}=random\;location$$
    end
end
for t=1:n3(n3= entire count of squirrels on normal trees moving to hickory trees)
    if $R_{3}\geq P_{dp}$
$$FS_{nt}^{new}=FS_{nt}^{old}+d_{g}G_{c}\left(FS_{ht}^{old}-FS_{nt}^{old}\right)$$
    else
$$FS_{nt}^{new}=random\;location$$
    end
end
$$S_{c}^{t}=\sqrt{\overset{n}{\underset{k--1}{\sum }}{\left(FS_{atk}^{t}-FS_{htk}\right)}^{2}},\;S_{c\;\min\;}=\frac{10B-6}{365^{Iter/\left(Iter_{\;\max\;}\right)/2.5}}$$
if $s_{c}^{t}<s_{c\;\min\;}$
$$FS_{nt}^{new}=FS_{L}+L\overset{´}{e}vy\left(n\right)\times \left(FS_{U}-FS_{L}\right)$$
end
Compute fitness value of novel places
$$f_{i}=f_{i}\left(FS_{i,1}^{new},FS_{i,2}^{new},\;\ldots ,FS_{i,n}^{new}\right),\;i=1,2,\;\ldots ,NP$$
Iter=Iter+1
end

The SSO algorithm derives a fitness function (FF) for reaching maximum classifier performance. It determines positive values for signifying the superior outcome of the candidate solutions. In this article, the reduction of the classifier error rate is the FF, as presented below in Equation (9):


(9) $$fitness\left(x_{i}\right)=ClassifierErrorRate\left(x_{i}\right)\;=\frac{number\;of\;misclassified\;samples}{Total\;number\;of\;samples}*100$$

### 3.4. Severity Extraction Process

Lexicons such as Sent WordNet and WordNet Affect are common lexicons that are utilized for extracting common sentiments of texts, for instance, movies and social reviews. The subjectivity lexicon has been utilized for extracting subjective expression in arguments or text statements. Several common and subjectivity lexicons have been changed in medicinal study to distinct healthcare tasks. A wide pharmaceutical lexicon has also progressed specifically to the biomedical and healthcare domains and has been used for extracting the sentiments of clinical and pharmaceutical text. It can extract the polarity of sentences by executing Sent WordNet, and the interface has been classified as low, moderate, or high levels, as dangerous and advantageous DDIs are dependent upon the polarity of candidate sentences.

## 4. Results and Discussion

**Figure 3:**
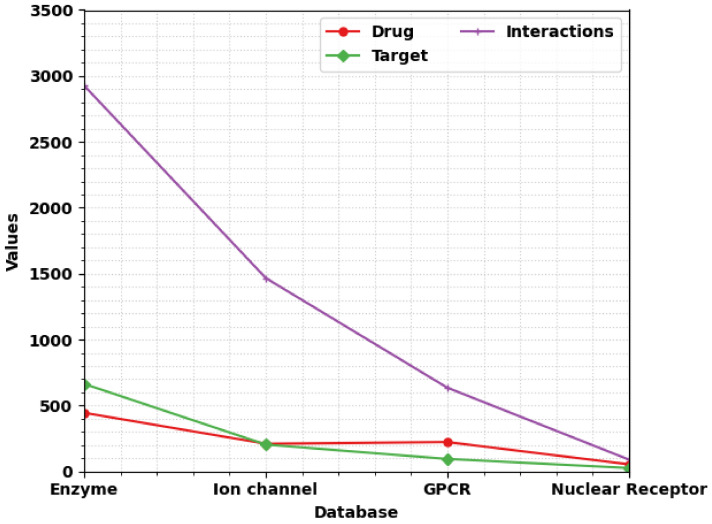
Sample distribution.

The experimental validation of the SSODL-DDIP technique was tested using drug target datasets [10,21]. We used four different datasets to examine the performance of the SSODL-DDIP technique. Table 1 presents the details of the datasets. The distribution of samples under drug, target, and interactions is given in Figure 3.

**Figure 4:**
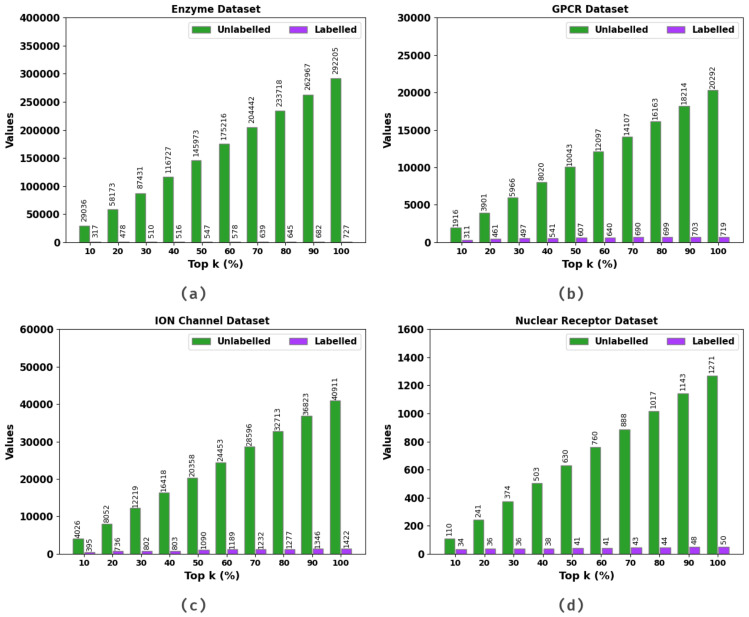
Result analysis of the SSODL-DDIP system: (**a**) Enzyme; (**b**) GPCR; (**c**) ion channel; (**d**) nuclear receptor.

Table 2 and Figure 4 present the performance of the SSODL-DDIP technique under unlabeled and labeled samples on the top k% values. The results indicate that the SSODL-DDIP technique effectively labeled the samples. For instance, on the top 10% of the enzyme dataset, the SSODL-DDIP technique labeled 317 samples under 29,036 unlabeled samples. Likewise, on the top 10% of the G protein-coupled receptors (GPCR) dataset, the SSODL-DDIP technique labeled 311 samples under 1916 unlabeled samples. Similarly, on the top 10% of the ion channel dataset, the SSODL-DDIP technique labeled 395 samples under 4026 unlabeled samples. Lastly, on the top 10% of the nuclear receptor dataset, the SSODL-DDIP technique labeled 34 samples under 110 unlabeled samples.

Table 3 presents the overall results of the area under the ROC curve (AUC) and the area under the precision-recall curve (AUPR) analysis of the SSODL-DDIP technique on four datasets.

**Figure 5:**
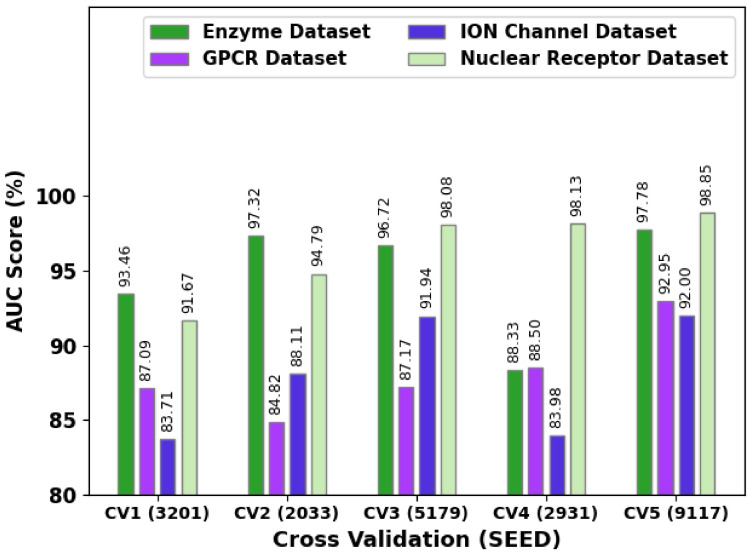
AUC analysis of the SSODL-DDIP technique under different CV_seed values.

Figure 5 shows the comprehensive AUC values of the SSODL-DDIP technique under different coefficient of variation (CV)_seed values. The figure shows that the SSODL-DDIP technique reached maximum AUC values under all datasets. For instance, on the enzyme dataset, the SSODL-DDIP technique attained higher AUC values of 93.46%, 97.32%, 96.72%, 88.33%, and 97.78% under CV_SEED values of 3201, 2033, 5179, 2931, and 9117, respectively. On the GPCR dataset, the SSODL-DDIP technique attained higher AUC values of 87.09%, 84.82%, 87.17%, 88.50%, and 92.95% under CV_SEED values of 3201, 2033, 5179, 2931, and 9117, respectively.

**Figure 6:**
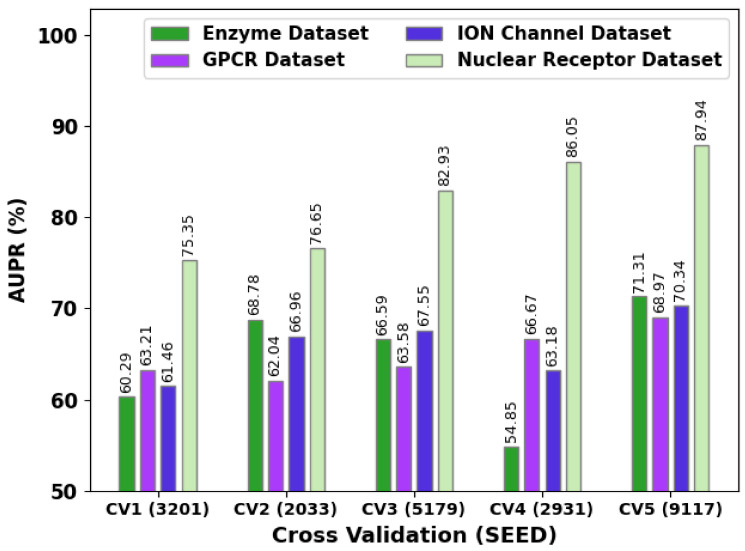
AUPR analysis of the SSODL-DDIP system under different CV_seed values.

Figure 6 presents the comprehensive AUPR values of the SSODL-DDIP technique under different CV_seed values. The figure implied that the SSODL-DDIP technique attained maximum AUPR values under all datasets. For example, on the enzyme dataset, the SSODL-DDIP technique attained higher AUPR values of 60.29%, 68.78%, 66.59%, 54.85%, and 71.31% under CV_SEED values of 3201, 2033, 5179, 2931, and 9117 respectively. On the GPCR dataset, the SSODL-DDIP technique attained higher AUPR values of 63.21%, 62.04%, 63.58%, 66.67%, and 68.97% under CV_SEED values of 3201, 2033, 5179, 2931, and 9117 respectively.

**Figure 7:**
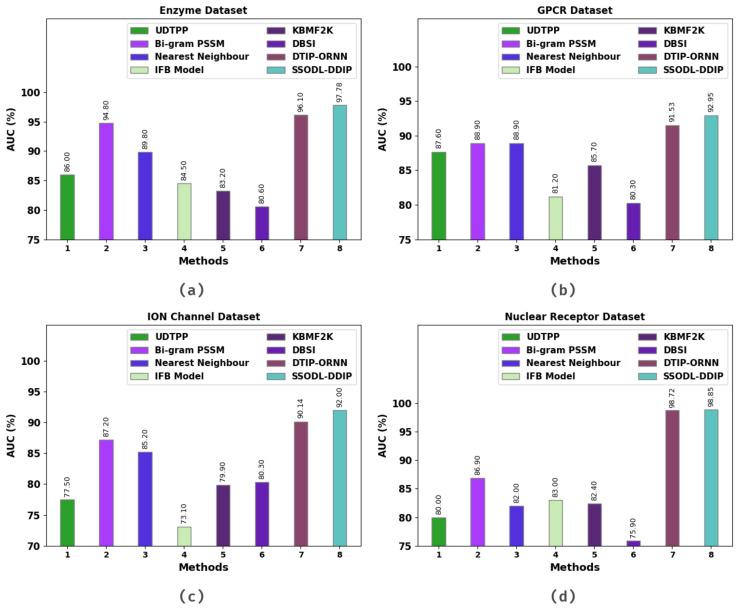
AUC analysis of the SSODL-DDIP technique: (**a**) Enzyme; (**b**) GPCR; (**c**) ion channel; (**d**) nuclear receptor.

Table 4 and Figure 7 show the results of a comparison study of the SSODL-DDIP technique on four datasets in terms of AUC [22,23,24,25]. The experimental values indicate that the SSODL-DDIP technique attained maximum AUC values under all datasets. For instance, on the enzyme dataset, the SSODL-DDIP technique attained a higher AUC value of 97.78%. In contrast, the bigram position-specific scoring matrix (PSSM), neural network (NN), IFB, kernelized Bayesian matrix factorization with twin kernels’ (KBMF2K), drug-based similarity inference (DBSI), and drug–target interaction prediction model using optimal recurrent neural network (DTIP-ORNN) technique attained lower AUC values of 86%, 94.80%, 89.80%, 84.50%, 83.20%, 80.60%, and 96.10% respectively. On the GPCR dataset, the SSODL-DDIP technique attained a higher AUC value of 92.95%. Conversely, the bigram PSSM, NN, IFB, KBMF2K, DBSI, and DTIP-ORNN technique attained lower AUC values of 86%, 87.60%, 88.90%, 88.90%, 81.20%, 85.70%, 80.30%, and 91.53%, respectively.

**Figure 8:**
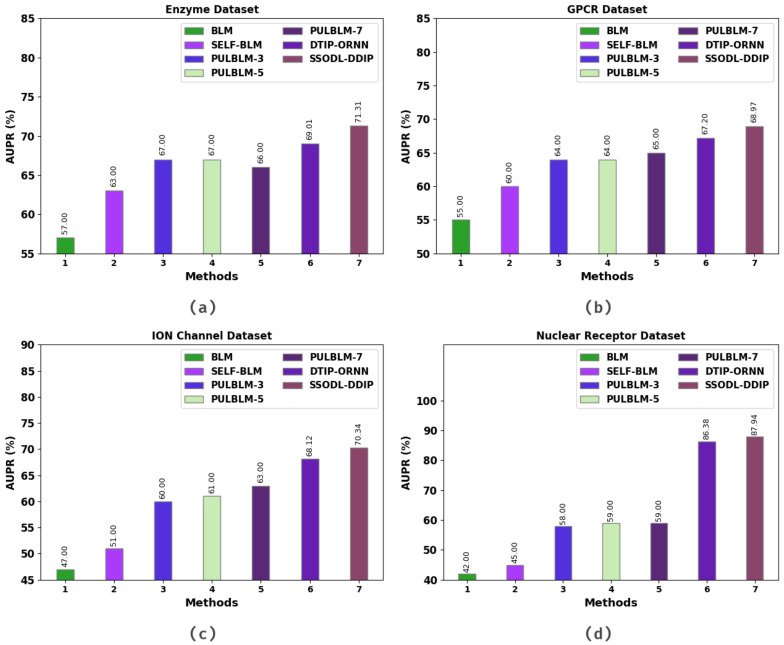
AUPR analysis of the SSODL-DDIP technique: (**a**) Enzyme; (**b**) GPCR; (**c**) ion channel; (**d**) nuclear receptor.

Table 5 and Figure 8 present a comparative inspection of the SSODL-DDIP technique on four datasets in terms of AUPR. The simulation values indicate that the SSODL-DDIP technique attained maximum AUPR values under all datasets. For instance, on the enzyme dataset, the SSODL-DDIP technique attained a higher AUPR value of 71.31%. In contrast, the bipartite local model (BLM), self-training support vector machine with BLM (SELF-BLM), positive-unlabeled learning with BLM (PULBLM)-3, PULBLM-5, PULBLM-7, and DTIP-ORNN technique attained lower AUPR values of 57.00%, 63.00%, 67.00%, 67.00%, 66.00%, and 69.01% respectively. In addition, on the GPCR dataset, the SSODL-DDIP technique attained a higher AUPR value of 68.97%. In contrast, the bigram BLM, SELF-BLM, PULBLM-3, PULBLM-5, PULBLM-7, and DTIP-ORNN technique attained lower AUPR values of 55.00%, 60.00%, 64.00%, 64.00%, 65.00%, and 67.20%, respectively. These results confirmed the effective DDI prediction results of the SSODL-DDIP technique.

## 5. Conclusions

In this study, we introduced a novel SSODL-DDIP technique for DDI predictions in big data environments. The presented SSODL-DDIP technique accurately determined the relationship and drug properties from various sources to make a prediction. In addition, the MLSTM-AE model was employed for the DDI prediction process. Furthermore, a lexicon-based approach was involved in determining the severity of interactions among the DDIs. To improve the prediction outcomes of the MLSTM-AE model, the SSO algorithm was adopted in this work. To assure better performance of the SSODL-DDIP technique, a wide range of simulations were performed. The experimental outcomes show the promising performance of the SSODL-DDIP technique over recent state-of-the-art methodologies. Thus, the SSODL-DDIP technique can be employed for improved DDI predictions. In the future, hybrid metaheuristics could be designed to improve the prediction performance. In addition, outlier detection and clustering techniques could be integrated to enhance the predictive results of the proposed model. 

## Figures and Tables

**Table 1:** Details on the datasets.

Dataset	Drug	Target	Interactions
Enzyme dataset	445	664	2926
Ion channel dataset	210	204	1467
GPCR dataset	223	95	635
Nuclear receptor dataset	54	26	90

**Table 2:** Analysis results of the SSODL-DDIP technique applied to distinct datasets.

Enzyme Dataset			GPCR Dataset		
Top k (%)	Unlabeled	Labeled	Top k (%)	Unlabeled	Labeled
10	29,036	317	10	1916	311
20	58,173	478	20	3901	461
30	87,431	510	30	5966	497
40	116,727	516	40	8020	541
50	145,973	547	50	10,043	607
60	175,216	578	60	12,097	640
70	204,442	639	70	14,107	690
80	233,718	645	80	16,163	699
90	262,967	682	90	18,214	703
100	292,205	727	100	20,292	719
**Ion Channel Dataset**			**Nuclear Receptor Dataset**		
**Top k (%)**	**Unlabeled**	**Labeled**	**Top k (%)**	**Unlabeled**	**Labeled**
10	4026	395	10	110	34
20	8052	736	20	241	36
30	12,219	802	30	374	36
40	16,418	803	40	503	38
50	20,358	1090	50	630	41
60	24,453	1189	60	760	41
70	28,596	1232	70	888	43
80	32,713	1277	80	1017	44
90	36,823	1346	90	1143	48
100	40,911	1422	100	1271	50

**Table 3:** AUC and AUPR analysis of the SSODL-DDIP system under distinct datasets.

Enzyme Dataset			GPCR Dataset		
CV_SEED	AUC	AUPR	CV_SEED	AUC	AUPR
3201	93.46	60.29	3201	87.09	63.21
2033	97.32	68.78	2033	84.82	62.04
5179	96.72	66.59	5179	87.17	63.58
2931	88.33	54.85	2931	88.50	66.67
9117	97.78	71.31	9117	92.95	68.97
**Ion Channel Dataset**			**Nuclear Receptor Dataset**		
**CV_SEED**	**AUC**	**AUPR**	**CV_SEED**	**AUC**	**AUPR**
3201	83.71	61.46	3201	91.67	75.35
2033	88.11	66.96	2033	94.79	76.65
5179	91.94	67.55	5179	98.08	82.93
2931	83.98	63.18	2931	98.13	86.05
9117	92.00	70.34	9117	98.85	87.94

**Table 4:** Comparative analysis of the SSODL-DDIP technique on different datasets in terms of AUC.

Methods	Enzyme	GPCR	ION Channel	Nuclear Receptor
UDTPP	86.00	87.60	77.50	80.00
Bi-gram PSSM	94.80	88.90	87.20	86.90
Nearest neighbor	89.80	88.90	85.20	82.00
IFB model	84.50	81.20	73.10	83.00
KBMF2K	83.20	85.70	79.90	82.40
DBSI	80.60	80.30	80.30	75.90
DTIP-ORNN	96.10	91.53	90.14	98.72
SSODL-DDIP	97.78	92.95	92.00	98.85

**Table 5:** Comparative analysis of the SSODL-DDIP technique on different datasets in terms of AUPR.

Methods	Enzyme	GPCR	ION Channel	Nuclear Receptor
BLM	57.00	55.00	47.00	42.00
SELF-BLM	63.00	60.00	51.00	45.00
PULBLM-3	67.00	64.00	60.00	58.00
PULBLM-5	67.00	64.00	61.00	59.00
PULBLM-7	66.00	65.00	63.00	59.00
DTIP-ORNN	69.01	67.20	68.12	86.38
SSODL-DDIP	71.31	68.97	70.34	87.94

## Data Availability

Data sharing is not applicable to this article as no datasets were generated during the current study.

## Abbreviations

**Abbreviation**	**Meaning**
DDI	Drug–drug interactions
ML	Machine learning
DL	Deep learning
SSODL-DDIP	Sparrow search optimization with deep learning-based DDI prediction
MLSTM-AE	Multilabel long short-term memory with an autoencoder
DM	Data mining
RBM	Restricted Boltzmann machines
CNN	Convolution neural networks
AE	Autoencoder
PU	Positive unlabeled
SVM	Support vector machine
GCN	Graph convolution network
DNN	Deep neural networks
KGNN	Knowledge graph neural network
KG	Knowledge graph
AMDE	Attention-related multidimensional feature encoders
SEV-DDI	Severity DDI
DANN-DDI	Deep attention neural network-related DDI
SFLLN	Sparse feature learning ensembled approach with linear neighborhood regularization
DP	Drug pairs
WEMB	Word embedding
LSTM	Long short-term memory
FCN	Fully connected networks
FC	Fully connected
FS	Flying Squirrels
GPCR	G protein-coupled receptors
AUPR	Area under the precision-recall curve
AUC	Area under the ROC Curve
CV	Coefficient of variation
PSSM	Position-specific scoring matrix
NN	Neural network
KBMF2K	Kernelized Bayesian matrix factorization with twin kernels
DBSI	drug-based similarity inference
DTIP-ORNN	Drug–target interaction prediction model using optimal recurrent neural network
BLM	Bipartite local model
SELF-BLM	Self-training support vector machine with BLM
PULBLM	Positive unlabeled learning with BLM
